# Lyapunov Exponents of a Discontinuous 4D Hyperchaotic System of Integer or Fractional Order

**DOI:** 10.3390/e20050337

**Published:** 2018-05-03

**Authors:** Marius-F. Danca

**Affiliations:** Romanian Institute of Science and Technology, 400487 Cluj-Napoca, Romania; danca@rist.ro

**Keywords:** fractional-order system, Caputo’s derivative, discontinuous initial value problem, continuous approximation, Lyapunov exponent

## Abstract

In this paper, the dynamics of local finite-time Lyapunov exponents of a 4D hyperchaotic system of integer or fractional order with a discontinuous right-hand side and as an initial value problem, are investigated graphically. It is shown that a discontinuous system of integer or fractional order cannot be numerically integrated using methods for continuous differential equations. A possible approach for discontinuous systems is presented. To integrate the initial value problem of fractional order or integer order, the discontinuous system is continuously approximated via Filippov’s regularization and Cellina’s Theorem. The Lyapunov exponents of the approximated system of integer or fractional order are represented as a function of two variables: as a function of two parameters, or as a function of the fractional order and one parameter, respectively. The obtained three-dimensional representation leads to comprehensive conclusions regarding the nature, differences and sign of the Lyapunov exponents in both integer order and fractional order cases.

## 1. Introduction

Systems with discontinuous right-hand side modeled as Initial Value Problems (IVPs) are mostly ideal, since switch-type functions like sgn are used, where the hysteresis or delay of the real switching operation is not considered, or the regularization represents a good approach for numerical integration of the underlying problems. Discontinuous functions can be found in two-dimensional mechanical systems such as systems with dry friction, oscillating systems combined with dry and viscous damping, forced vibrations, systems with stick and slip modes, brake processes with locking phases, control synthesis for uncertain systems, elastoplasticity, and also in game theory, optimization, control theory, calculus of variations, biological and physiological systems, electrical (chaotic) circuits, complex networks, power electronics, etc. (for examples, see [1,2,3] and references therein).

There are two main strategies to approach numerically discontinuous systems of integer order (IO): one strategy is to simply ignore the discontinuities (time stepping methods) and to rely on a local error estimator such that the error remains acceptably small, while the other strategy is to use a scalar event function $h:R^{n}\to R$, which defines the discontinuity $\Sigma =\{x\in R^{n}|h\left(x\right)=0\}$, to determine the intersection point as the new starting point for continuing the numerical solution (event-driven methods).

For numerical integration of discontinuous ordinary differential equations (ODEs) of IO, there exist dedicated numerical methods (see e.g., [4,5,6], or the survey [7]). However, note that a numerical method for discontinuous systems may become either inaccurate or inefficient, or both, in the discontinuity region. Moreover, the local truncation error analysis, which forms the basis of most step-size control techniques, fails if there is not sufficient local smoothness.

Note also that the numerical methods to solve a continuous ODE are faced with difficulties when the equation presents discontinuities on the right-hand side. Consider, for example, the Matlab integrator ode45. Jan Simon (from Matlab-related website [8,9]) pointed out that using this integrator or other routines for continuous systems, one can sometimes reach a final value, but this final value cannot be considered as a real “result” from the viewpoint of a scientist working in the field of numerical computations. For such systems, the numerical integration in the neighborhood of the discontinuity, using numerical methods for continuous systems, is a kind of measurement process based on an extremely large number of smaller and smaller steps, which cause round-off errors and local discretization errors. Also, the step-size control of the routine ode45 can lead to unexpected effects and the solver might integrate right over a discontinuity without noticing this. In these cases, the results have poor accuracies, which are highly doubtful especially in the neighborhoods of discontinuities. Thus, Matlab ode45 might reduce the step size to such a tiny value that the integration could take extremely long computational time to run while the accumulated rounding errors dominate the solutions. Similarly, in Mathematica, NDSolve cannot deal with discontinuities without special treatments.

Although fractional-order (FO) systems modeled with discontinuous functions have not been rigorously proved and analyzed, they could have better physical meaning for real systems. While the crossing of the solutions of continuous systems of FO on switching surfaces can be locally analyzed and then the whole dynamics can be composed by locally defined flow maps, for discontinuous systems of FO, however, this is not possible. In fact, for discontinuous systems of FO, transversally crossing or sliding solutions have not yet been analyzed. Moreover, the existing theory of Lyapunov Exponents (LEs) for classical dynamical systems remains to be generalized to discontinuous systems of IO, and then of FO.

On the other side, although there are numerical methods for FDEs (see, for example, [10,11] or [12] and references therein) and also for differential equations of IO with discontinuous right-hand sides, there are no numerical methods for FDEs with discontinuous right-hand sides. Consequently, continuously or smoothly modeling the underlying systems of IO, or FO, represents a possible challenge.

In [13,14], Benettin et al. proposed a Gram–Schmidt orthogonalization procedure to compute finite-time LEs for continuous systems of IO, as described in [15]. However, the algorithm is designed for continuous systems of IO only. Therefore, in this paper the LEs are numerically determined, after the considered IVP of IO or FO is continuously approximated. For the FO case, in [16] a Matlab code is presented to calculate the LEs of continuous systems of FO.

As generally accepted, a hyperchaotic system has two positive LEs, one null exponent along the flow and one negative exponent. However, this is not always the case; there exist 4D systems with more positive exponents [17,18,19] or even without zero exponents (see e.g., [20] and references therein). Therefore, representing graphically three-dimensionally the LEs, as a function of two parameters, or of one parameter and the fractional order, reveals that not only the numbers of positive, zero and negative exponents but also their dynamics, and allows an easier understanding and interpretation compared to the standard representation as a function of FO or parameter.

On the other side, determination of LEs of discontinuous systems of IO or FO requires the numerical integration of the underlying IVP, which cannot be realized with the classical methods for continuous differential equations of IO or FO, respectively.

LEs measure the average rate of divergence or convergence of orbits starting from nearby initial points. Therefore, they can be used to analyze the stability of limits sets and to check the sensitive dependence on initial conditions, that is, the presence of potential chaotic attractors.

In numerical experiments, one can only consider finite-time numerically computed values of LEs, which can differ significantly (e.g., if the considered trajectory belongs to a transient chaotic set). Therefore, the numerically computed LEs are considered as local finite-time LEs.

The existence of local finite-time LEs of a discontinuous hyperchaotic system of IO or FO, will be exemplified graphically on the system described by the following commensurate FO differential equations:

(1) $$\begin{array}{c} \frac{d^{q}}{dt^{q}}x_{1}=a\left(x_{2}-x_{1}\right),\\ \frac{d^{q}}{dt^{q}}x_{2}=x_{1}x_{3}-x_{1}x_{4},\\ \frac{d^{q}}{dt^{q}}x_{3}=b-x_{1}x_{2},\\ \frac{d^{q}}{dt^{q}}x_{4}=c\mathrm{sgn}\left(x_{3}\right)-kx_{4}, \end{array}$$

where 0<q≤1, and parameters a,b,c and *k* are real. Given the variations of each parameter generates rich dynamics, they can be considered as bifurcation parameters [21,22].

If q=1, $\frac{d^{q}}{dt^{q}}$ stands for the usual derivative of IO: $\frac{d^{q}}{dt^{q}}x=\frac{d}{dt}x:=\dot{x}$, while for q∈(0,1), $\frac{d^{q}}{dt^{q}}$ represents Caputo’s derivative with starting point 0, denoted, $D_{*}^{q}$, $\frac{d^{q}}{dt^{q}}x=D_{*}^{q}$ [23,24,25]. The advantage of using Caputo’s derivative is it allows the use of initial conditions in the standard form, $x\left(0\right)=x_{0}$, as for the IO case.

The IO variant of this system yielded from a 3D Sprott system of IO [21] is presented in [22].

Being a discontinuous system, it cannot be numerically integrated with the common routines designed for continuous ODEs of IO or FO (see e.g., [19]). To allow the study of numerical LEs, a possible continuous regularization of the right-hand side to overcome the discontinuity problem is presented in this paper.

The paper is organized as follows. Section 2 presents the approach of discontinuous differential equations of IO or FO; Section 3 deals with the three-dimensional graphical representation of LEs. Finally, the Conclusion section ends the paper.

## 2. Numerical Integration of the IVP (1)

System (1) belongs to the class of systems with discontinuous right-hand side modeled by the following FO IVP of commensurate order *q*:

(2) $$\frac{d^{q}}{dt^{q}}x\left(t\right)=f\left(x\left(t\right)\right),\;\;x\left(0\right)=x_{0},\;\;\;t\in I=\left[0,\infty \right),$$

where $f:R^{n}\to R^{n}$ is a function in the following form:

(3) f(x(t))=g(x(t))+A(x(t))s(x(t)),

and $g:R^{n}\to R^{n}$ is a continuous function, $s:R^{n}\to R^{n}$, $s\left(x\right)={\left(s_{1}\left(x_{1}\right),s_{2}\left(x_{2}\right),...,s_{n}\left(x_{n}\right)\right)}^{T}$ a discontinuous function, with $s_{i}:R\to R$, i=1,2,…,n, piece-wise constant functions (e.g., sgn function) and *A* a square matrix of functions.

Being an autonomous system, hereafter the time variable *t* will be suppressed.

For system (1):

$$g\left(x\right)=\left(\begin{array}{c} a\left(x_{2}-x_{1}\right)\\ x_{1}x_{3}-x_{1}x_{4}\\ b-x_{1}x_{2}\\ -kx_{4} \end{array}\right),\;\;s\left(x\left(t\right)\right)=\left(\begin{array}{c} \mathrm{sgn}(x_{1})\\ \mathrm{sgn}(x_{2})\\ \mathrm{sgn}(x_{3})\\ \mathrm{sgn}(x_{4}) \end{array}\right)\;\;\;\mathrm{and}\;\;A\left(x\right)=\left(\begin{array}{cccc} 0 & 0 & 0 & 0\\ 0 & 0 & 0 & 0\\ 0 & 0 & 0 & 0\\ 0 & 0 & c & 0 \end{array}\right).$$

To understand the complexity of the numerical integration of the discontinuous problem (2), consider for example the simple case q=1, and the example $\dot{x}=1-2\mathrm{sgn}\left(x\right)$, $x\left(0\right)=x_{0}$ [26]. The equation has no classical (continuously differentiable) solutions. Thus, if $x_{0}\neq 0$, a solution is $x\left(t\right)=3t+x_{0}$, for x<0 and $x\left(t\right)=-t+x_{0}$, if x>0. However, as *t* increases, in the plane (t,x) these solutions tend to the line x=0 and tend to remain on this line but never leave it upwards or downwards. Moreover, once a solution has arrived on the line x=0 it cannot move along this line, because the solution x(t)=0 does not satisfy the equation in the usual sense: its derivative is $\dot{x}\left(t\right)=0$, while the function on the right-hand side gives 1−2sgn(0)=1.

On the other side, the IVP of FO, $D_{*}^{q}x=2-3\mathrm{sgn}\left(x\right)$, $x\left(0\right)=x_{0}$ [16], has no classical solutions starting from any point $x_{0}$ (see [27] for solutions to FDEs). Thus, for $x=x_{0}=0$, there is no solution since $D_{*}^{q}0=0\neq 2-3\mathrm{sgn}\left(0\right)$. For $x_{0}>0$, even there exists a solution, it exists only on the interval $\left[0,T^{\prime }\right)$ with $T^{\prime }={\left(\Gamma \left(1+q\right)x_{0}\right)}^{1/q}$, where Γ is the Gamma function. In this case, the solution is $x\left(t\right)=x_{0}-t^{q}/\Gamma \left(1+q\right)$, which cannot be extended to any interval larger than $\left[0,T^{\prime }\right)$. If $x_{0}<0$, there also exists some $T^{\prime \prime }={\left(\Gamma \left(1+q\right)x_{0}/5\right)}^{1/q}$, and the solution $x\left(t\right)=x_{0}+5t^{q}/\Gamma (1+q)$ exists but only on $\left[0,T^{\prime \prime }\right)$. Also, although these solutions tend to the line x=0, they cannot be extended along this line.

Thus, a discontinuous IVP (of IO or FO) might have no classical solutions (see [26,28] for the IO case).

In other words, using dedicated numerical methods for continuous IO or FO differential equations, such as Matlab integrators or the Adams-Bashforth-Moulton (ABM) method for FO differential equations respectively, might only give apparently correct results (see [29] for a correct numerically integration approach to this kind of discontinuous problems).

A simple way to deal with discontinuities, which can be easily implemented computationally, is to approximate the discontinuous functions *s* by continuous functions.

The possibility to approximate the right-hand side of (2) is ensured by the following theorem.

**Theorem** **1** ([19])**.** *If g is continuous, then system *(2)*–*(3)* with q≤1 can be continuously approximated by the following IVP:*

$$\frac{d^{q}}{dt^{q}}x=\tilde{f}\left(x\right),\;\;\;x\left(0\right)=x_{0},\;\;\;t\in I,$$

*where $\tilde{f}$ is a continuous approximation of f. Furthermore, if g is smooth, then $\tilde{f}$ is smooth.*

The proof is based on the Fillipov regularization [26], which allows to transform the discontinuous problem into a set-valued IVP (see e.g., [28]). Next, based on Cellina’s Theorem ([28] (Theorem 1, p. 84), or [30] (Theorem 9.2.1, pp. 358–359)), the set-valued problem admits a continuous approximation $\tilde{f}$ (see the proof in [19]). The approximation given by Cellina’s Theorem is smooth and, if *g* is also smooth, $\tilde{f}$ is smooth.

**Figure 1:**
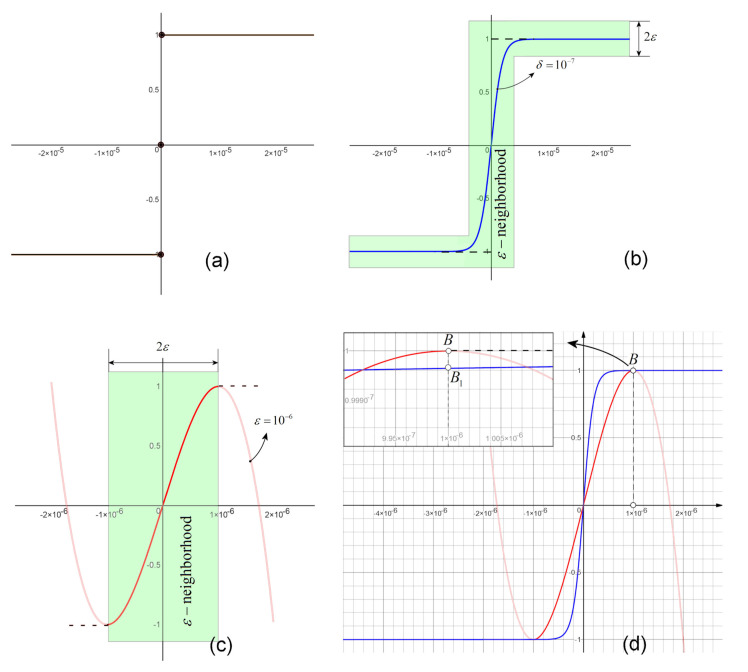
(**a**) Graph of sgn function; (**b**) Graph of the global approximation of sgn: $\frac{2}{1+e^{-\frac{x}{\delta }}}-1$ for $\delta =10^{-7}$, included in an ε-neighborhood; (**c**) Graph of the local approximation of sgn: $-\frac{1}{2\varepsilon^{3}}x^{3}+\frac{3}{2\varepsilon }x$ for $\varepsilon =10^{-6}$, defined in the ε-neighborhood of the discontinuity x=0; (**d**) Superimposed graphs of local and global approximation. The magnified rectangle reveals that the global approximation reaches only asymptotically the branches of sgn.

Consider, for simplicity, the case of sgn function (Figure 1a). The approximation can be realized either within an ε-band centered on the branches of the sgn function, i.e., *global approximation* defined along the entire branches (light-green ε-band in Figure 1b), or only on a small enough ε-neighborhood of the discontinuity, i.e., *local approximation*, defined on a small enough ε-neighborhoods of x=0 (Figure 1c).

One of the best candidates for the local approximation is the following sigmoid-like function [19]:

(4) $$\tilde{\mathrm{sgn}}\left(x\right)=\frac{2}{1+e^{-\frac{x}{\delta }}}-1\approx \mathrm{sgn}\left(x\right),\;\;\;x\in R,$$

where δ is a parameter which controls the slope of the function and, implicitly, the ε-neighborhood size (see Figure 1b for $\delta =10^{-7}$).

As global approximation of the sgn function, the cubic polynomials represent a possible choice:

(5) $${\tilde{\mathrm{sgn}}}_{\varepsilon }\left(x\right)=-\frac{1}{2\varepsilon^{3}}x^{3}+\frac{3}{2\varepsilon }x\approx \mathrm{sgn}\left(x\right),\;\;\;x\in \left[-\varepsilon ,\varepsilon \right],$$

which is smoothed at the “gluing” points ±ε [19].

**Remark** **1.** While the global approximation *(4)* is less computer time consuming and can be simply implemented (by replacing the sgn function), the local approximation *(5)* requires relatively longer computer time and also the interception of the ε-neighborhood crossing, when sgn must be replaced. On the other side, as shown in [19], the local approximation offers a better approximation (see Figure 1d, where one can see that, compared to the local approximation, the global approximation only tends to reach (asymptotically) the horizontal branches of the sgn function). Also, while ε in the local approximation *(5)* represents the size of the ε-approximation, the relation between ε size and δ in the global approximation is rater complicated and difficult to find.

In this paper, the local approximation is adopted with $\varepsilon =1\times 10^{-6}$.

Once the system (2) is approximated, the underlying equations (of IO or FO) can be numerically integrated with some standard method for continuous differential equations, e.g., Matlab ode45, or the predictor-corrector multi-step Adams-Bashforth-Moulton (ABM) method for FO [10].

## 3. Graphical Analysis of Lyapuynov Exponents of System (1)

Assume that *g* in (2) is smooth. Then, $\tilde{f}$ is also smooth (Theorem 1) and, for the IO case, the existence of LEs is ensured. The existence of the variational equations, which determine the LEs, is given by the following theorem.

**Theorem** **2** ([16,19])**.** *The system *(1)*, with q<1, has well-defined LEs.*

Sketch of the proof: Under continuity assumption on *g*, and after the continuous (smooth) approximation of *f*, there exists a flow $\phi :I\times R^{n}\to R^{n}$ satisfying $D_{*}^{q}\phi \left(t,x_{0}\right)=\tilde{f}\left(\phi \left(t,x_{0}\right)\right)$, $\phi \left(0,x_{0}\right)=x_{0}$, for t∈I. Then, [31] (Theorem 2) applies.

To determine the spectrum of LEs for q∈(0,1], the Benettin algorithm is utilized (see the Matlab code for LEs of continuous systems of FO in [32]).

If the system is of IO, suppose it depends on at least two parameters.

Usually, the underlying LEs are determined graphically as one-variable function of one parameter, or of the FO, the graphs being curves. However, the graphical interpretation of LEs can be dramatically improved if the LEs are considered as functions of two variables: two parameters or one parameter and the FO. In these cases, the results are three-dimensional surfaces.

In order to determine numerically the LEs of the system (1) and representing the underlying LEs surfaces, in this paper the locally approximation (5) is utilized and the approximated system becomes a smooth system:

(6) $$\begin{array}{c} \frac{d^{q}}{dt^{q}}{x_{1}}_{1}=a\left(x_{2}-x_{1}\right),\\ \frac{d^{q}}{dt^{q}}x_{2}=x_{1}x_{3}-x_{1}x_{4},\\ \frac{d^{q}}{dt^{q}}x_{3}=b-x_{1}x_{2},\\ \frac{d^{q}}{dt^{q}}x_{4}=\left\{\begin{array}{c} c\mathrm{sgn}\left(x_{3}\right)-kx_{4},\;\;\mathrm{if}\;\;x_{3}\notin \left(-\varepsilon ,\varepsilon ,\right)\\ {c\tilde{\mathrm{sgn}}}_{\varepsilon }\left(x_{3}\right)-kx_{4},\;\;\mathrm{if}\;\;x_{3}\in \left[-\varepsilon ,\varepsilon \right]. \end{array}\right. \end{array}$$

As known, the LEs exponents are time-averaged along some typical trajectory in the phase space. Also, the computation over a relative longer time interval allows a more complete visualization of a chaotic attractor, with damage to the results accuracy. In this paper, the integration time interval is I=[0,250]. In order to avoid the interplay between different attractors (the system presents multistability, i.e., coexistence of attractors [17]), to remain focusing on the same attractor, same initial condition (0.1,0.1,0.1,0.1) is used. However, somewhat different initial conditions, chosen in the neighborhood of this point, give slightly similar results.

A “zero” LE is considered here as a real number with at least two first zero decimals, i.e., determined with errors less than $1\times 10^{-3}$ [20,33,34].

Let the LEs ordered as follows: $\lambda_{1}\geq \lambda_{2}\geq \lambda_{3}\geq \lambda_{4}$. The graphical representation of these LEs will be given in the space $\left(p_{1},p_{2},\lambda \right)$, where $p_{1,2}$ are either two parameters (in this paper, *a*, *b*, *c* or *k*) or one parameter and the FO *q*, and λ representing the values of LEs. Therefore, a zero LE represents graphically a point $\left(p_{1},p_{2},0\right)$, i.e., a point lying on the plane λ=0, while a positive or negative LE is represented by a point $\left(p_{1},p_{2},\lambda \right)$ with λ>0 or λ<0, respectively.

### 3.1. The Integer Case

**Figure 2:**
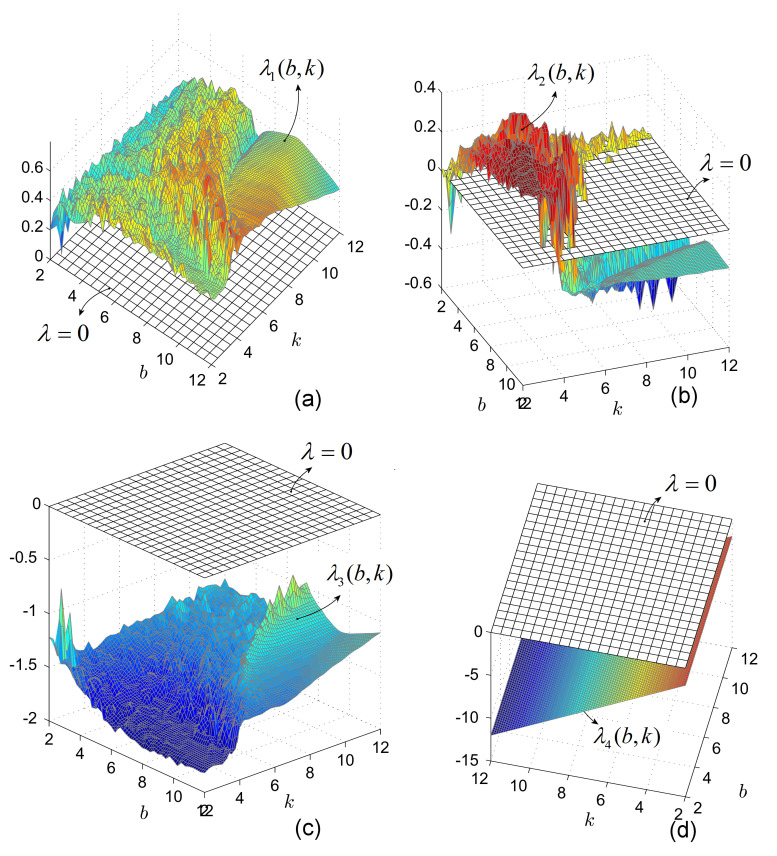
Graphs of LEs of system (6) of IO, represented as surfaces depending on the parameters *b* and *k*. The horizontal plane, λ=0, reveals the zero LEs. (**a**) Surface representing the evolution of $\lambda_{1}$; (**b**) Surface representing the evolution of $\lambda_{2}$; (**c**) Surface representing the evolution of $\lambda_{3}$; (**d**) Surface representing the evolution of $\lambda_{4}$.

Consider the IO case, i.e., the approximated system (6) with $\frac{d^{q}}{dt^{q}}x=\dot{x}$, and let a=1, c=9 and *b* and *k* be variable parameters (other possible combinations can be treated similarly). The numerical integration is realized with the Matlab ode45 routine and the obtained LEs are considered as functions of *b* and *k*, $\lambda_{i}\left(b,k\right)$, i=1,2,3,4, for (b,k) belonging to the lattice [2,12]×[2,12]. The results are plotted for clarity, in separated figures (Figure 2a–d).

The exponent $\lambda_{4}$ being negative, hereafter for clarity only $\lambda_{i}$, i=1,2,3 are considered.

**Figure 3:**
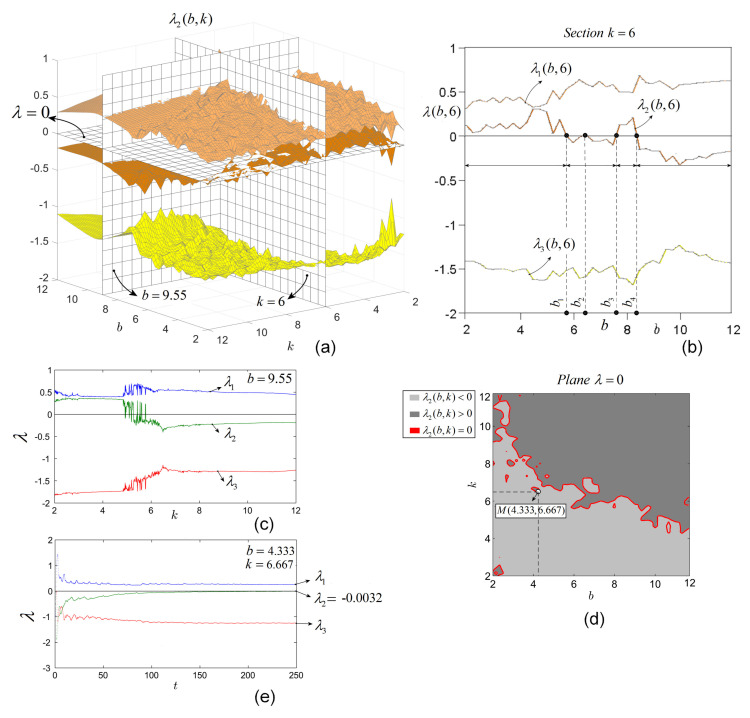
(**a**) First three overplotted LE surfaces of system (6) of IO; (**b**) First three LEs obtained by graphical section of the surfaces of LEs with the vertical plane k=6 (see also Figure 3a). Values $b_{1}-b_{4}$ correspond to the zero LE ($\lambda_{2}=0$); (**c**) The first three LEs as function of *k*, obtained numerically for b=9.55 (see, for comparison, the vertical section b=9.55 in Figure 3a); (**d**) Zero LE curves (red plot) obtained by sectioning the surface $\lambda_{2}\left(b,k\right)$ withe plane λ=0, containing the values (b,k) for which $\lambda_{2}=0$; (**e**) The first three LEs corresponding to (b,k)=(4.333,6.667) (point *M* in Figure 3d), revealing that $\lambda_{2}=0(-3\times 10^{-3}$).

As can be seen, for all *b* and *k*, there exist at least one positive LE, $\lambda_{1}>0$, and at least two negative $\lambda_{3,4}<0$. Therefore, the only exponent for which function of *b* and *k* can be zero or change sign, is $\lambda_{2}$. For a better understanding, consider Figure 3a. To obtain an usual representation of LEs, e.g., as function of the parameter *b* for a fixed value of *k* (e.g., k=6), one can consider a vertical section (perpendicular to the plane (b,k) and parallel with axis *b*), through k=6 (Figure 3a,b). Similarly, one can obtain the LEs for fixed *b* and k∈[2,12]. For example, LEs obtained numerically as function on *k* for b=9.55 are represented in Figure 3c (see the graphical section in Figure 3a, with b=9.55).

From the section with k=6 and b∈[2,12] (Figure 3b), one can see that, for $b\in \left[2,b_{1}\right]\cup \left[b_{3},b_{4}\right]$, the system admits two positive LEs, while for the other values of *b* the system has only a single positive LE. Regarding the zero LEs, even this section reveals that there exist few isolated values of *b*, for which the system admits zero LE ($b_{1}$, $b_{2}$, $b_{3}$ and $b_{4}$) if one crosses the surface of $\lambda_{2}$ with the plane λ=0 and it can be seen that the system actually has infinitely many values (points) (b,k)∈[2,12]×[2,12] for which $\lambda_{2}=0$. These points are situated on continuous “zero curves” (red plot) in Figure 3d (dark gray represents the points where $\lambda_{2}>0$, while light gray represents the points with $\lambda_{2}<0$). For example, for the point M(4.333,6.667), the LEs plotted as function of time (Figure 3e) show that $\lambda_{3}=-3\times 10^{-3}$.

This property seems to be a general characteristic of discontinuous systems and is different from the case of continuous systems of FO, where the zero LEs are situated on surfaces, not curves [19].

### 3.2. The Fractional-Order Case

Consider system (6) of FO, with a=1, b=1, c=9, and *k* and q∈(0,1) variables (similarly, one can obtain the other possible combinations). LEs are obtained with the Matlab code presented in [16].

**Figure 4:**
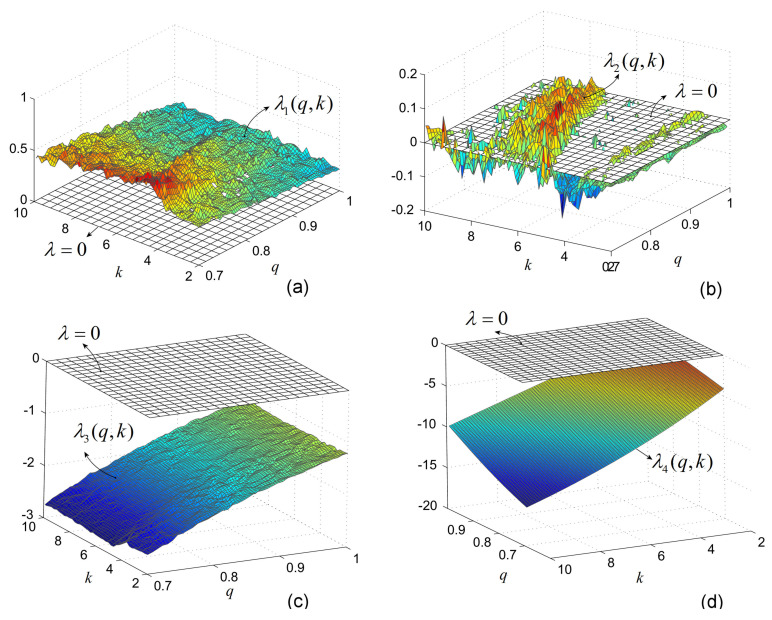
Graphs of LEs of the approximated system (6) of FO, represented as surfaces depending on the parameters *q* and *k*. The horizontal plane λ=0 reveals the zero LEs. (**a**) Surface representing the evolution of $\lambda_{1}$; (**b**) Surface representing the evolution of $\lambda_{2}$; (**c**) Surface representing the evolution of $\lambda_{3}$; (**d**) Surface representing the evolution of $\lambda_{4}$.

Let us consider the most interesting case of *q* values close to 1, which generate rich dynamics (as known, for lower values of *q*, chaos vanishes generally). Following the same procedure as for IO, the obtained LE surfaces for q∈(0.7,1) and k∈([2,10], are plotted separately in Figure 4a–d. As expected, similarly to the integer case, a single LE, $\lambda_{2}$, is potentially zero.

Again, for clarity, only consider the first three LEs.

**Figure 5:**
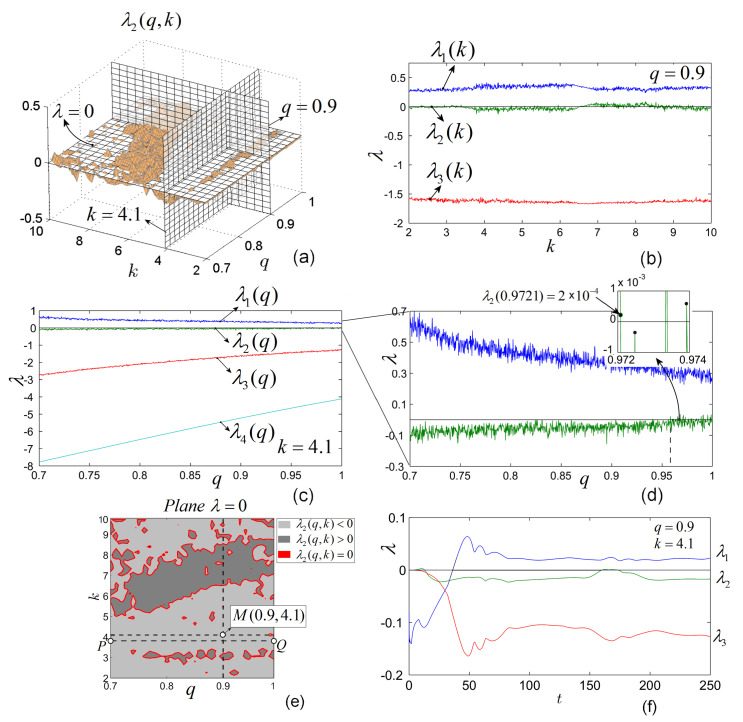
(**a**) Graph of the surface of the second LE of system (6) of FO; (**b**) First three LEs obtained numerically for q=0.9 (compare with the graphical vertical section q=0.9 in Figure 5a); (**c**) The first three LEs obtained numerically for k=4.1 as function of *q* (see also the vertical section k=4.1 in Figure 5a); (**d**) The magnified image of the first two LEs revealing the fact that there exists zero LE ($\lambda_{2}$) only for q>0.95; (**e**) Zero curves (red plot) obtained by sectioning the surface $\lambda_{2}\left(b,k\right)$ withe plane λ=0, containing the values (q,k) for which $\lambda_{2}=0$; (**f**) LEs corresponding to q=0.9 and k=4.1 (point *M* in Figure 5e). For these values of (q,k), there are no zero LEs.

For q=0.9 (see the vertical section with the plane q=0.9 in Figure 5a), the numerically determined LEs spectrum is plotted in Figure 5b (compare with the vertical section through q=0.9 in Figure 5a). The figure reveals that there are values of parameter *k* for which $\lambda_{2}$ is either zero, positive or negative.

Apparently for k=4.1, there is no zero LE (the surface of $\lambda_{2}$ does not cross the plane λ=0 for k=4.1, Figure 5a) and LEs have the signs (+,−,−,−). However, by a careful analysis, if one considers the vertical section with the plane k=4.1 (Figure 5c), the dynamics of LEs as function of *q* reveals that, for *q* close to 1 (q>0.95), there exists zero LE (see the zoom in Figure 5d, where the detail shows that there exists zero $\lambda_{2}$ determined with errors smaller than $1\times 10^{-3}$, $\lambda_{2}=2\times 10^{-4}$) (note that, in the zoomed image, the calculated values of LE are represented by circles, while in between the circles are obtained by linear interpolation).

Similarly, one can determine the zero curves formed by points (q,k) in the plane λ=0 for which there exist zero LEs ($\lambda_{2}$) (Figure 5e).

It is interesting to see that there are regions (surfaces) in the plane (q,k) where there does not exist zero LEs. Thus, for example for the point M(0.9,4.1) (Figure 5e), the LEs are different from zero (see Figure 5f for the first three LEs). Moreover, in this case, when the signs of LEs are (+,−,−,−), it persists for all q∈(0.7,1) if *k* is smaller and close to k=4 (see line segment PQ).

## 4. Conclusions

In this paper we proposed the three-dimensional representation of the local finite-time LEs spectrum of a discontinuous dynamical system of IO or FO.

The example of a hyperchaotic discontinuous 4D system of IO or FO depending on several parameters is considered.

To be numerically integrated, the system is continuously and smoothly approximated. In this way, the system can be correctly integrated by using the standard numerical integrators for IO or FO.

The evolution of the LEs is represented as a two-dimensional function of two variables: one of the parameters and the FO, is more suggestive and comprehensive compared to the classical representation of LEs as function of a parameter or of the FO. In this way, the considered hyperchaotic system is found to have always one positive LE or, depending on parameters or the order, two positive LEs. Therefore, for all considered parameter values of *b* and *k*, or FO *q* and *k*, the system is at least chaotic (one positive LE, $\lambda_{1}>0$) or even hyperchaotic (two positive LEs, $\lambda_{1,2}>0$).

Also, in the plane λ=0, there exist continuous zero curves of points (b,k) (in the case of IO system), or (q,k) (in the case of FO system), for which there exist zero LEs. Thus, the following signs of LEs are possible: (+,−,−,−), (+,0,−,−) or (+,+,−,−). Therefore, for the case of hyperchaotic systems, the standard characterization as systems with two positive LEs seems not being an adequate definition.

Another useful utilization of the three-dimensional representation of LEs is that it reveals a possible general property: discontinuous systems have zero LEs for parameters $\left(p_{1},p_{2}\right)$ (in the case of IO systems with two parameters) or (p,q) (in the case of systems of FO with a single parameter) situated along continuous curves, not on surfaces, unlike the case of continuous systems of IO or FO (see [19]).

The possibility to choose graphically the parameters or the FO, such that the considered system has a larger number of positive LEs, can be useful for secure communication where generating fractional-order hyperchaotic systems with a desired number of positive LEs is an open and important problem.

Another advantage of this kind of representation is in using only one-dimensional representation (as function on a single parameter or on the FO), it is impossible to conclude about the existence of zero LEs, or the number of positive LEs, while the three-dimensional representation allows suggestive visualization of the dynamics of the LEs.
